# Evaluation of mandibular functional limitation in patients with temporomandibular joint disorders and the relationship with other psychosocial factors

**DOI:** 10.1371/journal.pone.0334508

**Published:** 2025-10-24

**Authors:** Nguyen Ngoc Hoa, Toan Do, Tran Thai Binh, To Thanh Dong, Tran Thi Minh Quyen, Viet Hai Hoang

**Affiliations:** 1 School of Dentistry, Hanoi Medical University, Dong Da, Hanoi, Vietnam; 2 Department of Research Methodology and Biostatistics, School of Preventive Medicine and Public Health, Hanoi Medical University, Dong Da, Hanoi, Vietnam; 3 108 Military Central Hospital, Hai Ba Trung, Hanoi, Vietnam; 4 Department of Mathematics and Informatics, Hanoi Medical University, Dong Da, Hanoi, Vietnam; 5 Faculty of Traditional Medicine, Hanoi Medical University, Dong Da, Hanoi, Vietnam; University of Florida Jacksonville, UNITED STATES OF AMERICA

## Abstract

**Introduction:**

Temporomandibular joint disorders (TMDs) are a common source of orofacial pain and dysfunction, often characterized by myalgia, restricted mouth opening, and limitations in mandibular movement. This study aims to evaluate the extent of mandibular functional limitation across different TMD subgroups and its relationship with anxiety and depression.

**Methods:**

A cross-sectional descriptive study was conducted on 120 patients diagnosed with temporomandibular joint disorders according to the Diagnostic Criteria for TMDs (DC/TMD). Participants completed self-report questionnaires assessing the Jaw Functional Limitation Scale-8 (JFLS-8), Visual Analog Scale (VAS), Patient Health Questionnaire-9 (PHQ-9), General Anxiety Disorder-7 (GAD-7), Graded Chronic Pain Scale (GCPS), and Oral Behaviors Checklist (OBC). The Mann-Whitney U and Kruskal-Wallis tests were used to compare means between groups for non-normally distributed data. The relationship between JFLS-8 and other variables was assessed using linear regression. Statistical significance was set at **p* *< .05.

**Results:**

Chewing hard foods is the most affected activity in the arthralgia group (7.25 ± 1.91), while swallowing is the least affected activity (0.13 ± 0.35). Statistically significant differences were found among TMJ disorders for chewing tough food, chewing chicken, opening wide enough to drink from a cup, and yawning (**p* *< **.05). Disc displacement without reduction, with a limited opening subgroup, exhibited the highest JFLS-8 scores for chewing and mouth-opening-related activities. JFLS-8 is correlated with the PHQ-9, GAD-7, GCPS (**p* *< **.05).

**Conclusions:**

Significant variations in mandibular functional limitation were observed across TMD subgroups. The severity of these limitations demonstrated a positive correlation with levels of depression, anxiety, and chronic pain.

## Introduction

Temporomandibular disorders (TMDs) are associated with a range of clinical presentations, potentially resulting in functional limitations of the masticatory system and/or adverse psychosocial effects, including reduced quality of life [1]. The prevalence of TMDs was significantly higher in South America (47%) compared to Asia (33%) and Europe (29%), indicating a notable geographical variation in TMD occurrence [2]. TMD prevalence in children and adolescents was 18% and 17% among adults [3]. Functional limitations and disabilities associated with TMD are closely linked to underlying organ-level impairments and pathophysiological processes. A direct correlation exists between TMD symptom severity and the degree of functional limitation and disability. Consequently, integrating clinical findings with a thorough assessment of functional impact is crucial for optimizing TMD diagnosis and treatment strategies [4].

The Diagnostic Criteria for Temporomandibular Disorders (DC/TMD) employs a biopsychosocial model with established validity and reliability and is widely used in diagnosing and managing TMD. The DC/TMD guidelines recommend the Jaw Functional Limitation Scale (JFLS) as a reliable screening tool for assessing limitations in mandibular function related to mastication, mobility, verbal expression, and emotional expression. Axis I of the DC/TMD describes 10 commonly encountered diagnoses of temporomandibular joint disorders, divided into pain-related disorders (myalgia, myofascial pain with referral, arthralgia, headache attributed to TMD) and intra-articular temporomandibular disorders (disc displacement with reduction, disc displacement with reduction with intermittent locking, disc displacement without reduction with limited opening, disc displacement without reduction without limited opening, degenerative joint disease, subluxation) [5,6].

Traditional assessments of jaw function in TMDs and disc displacement research have focused on quantitative parameters such as pain, maximal mouth opening, and lateral range of motion [7,8]. While quantitative parameters provide valuable information, a comprehensive assessment of jaw function must also consider its impact on daily life. Activities such as eating, drinking, laughing, and speaking, all essential for social engagement, rely on proper jaw function. Therefore, evaluating the social aspect of jaw function using the questions in the JFLS-8 questionnaire in this study was considered important, as it is a suitable tool. The questionnaire allowed us to evaluate the limitations in jaw function experienced by patients with TMDs more comprehensively [9].

Clinical symptoms in TMD patients are closely related to functional limitation and disability [10]. A higher degree of functional jaw limitations was associated with an increasing probability of a positive screening outcome for functional jaw disturbances [4]. Deterioration of clinical symptoms in TMD patients leads to increased functional limitation and disability [10]. The values of the mobility of the mandible in the transverse plane and the associated acoustic symptoms assessed in the physical examination showed no correlation pattern with any other variables [11].

Compared to healthy controls, Karacayli et al. found that patients with chronic TMD pain and disc displacement with reduction exhibited greater limitations in jaw function, affecting activities such as smiling, cleaning their teeth or face, and speaking [12]. However, Sophie Lau Rui Han’s findings indicated a lack of significant correlation between temporomandibular joint effusion and TMJ pain, disc displacement, or jaw functional limitation [13]. A cross-sectional study found no association between TMJ intra-articular status and the TMD effect represented by pain, jaw function, and disability [14].

Most studies have primarily assessed mandibular functional limitations within pain-based subgroups of TMDs [15,16]. However, the relationship between specific TMD subgroups, particularly the common diagnoses described in Axis I of the Diagnostic Criteria for Temporomandibular Disorders (DC/TMD), and individual functional activities remains a subject of debate. TMD patients experienced impaired food intake ability [17]. Individuals with disc displacement (with or without reduction) demonstrated greater functional limitations compared to those with myofascial pain. Dietary factors and eating habits should be considered in the clinical management of patients with different TMD subtypes [17]. Myofascial pain with referral demonstrates a significant association with limitations in mastication, mandibular mobility, verbal and emotional communication, and overall functional status as assessed by the JFLS-20. Approximately 40% of patients with myofascial pain with referral experience jaw functional limitations [18].

Data from various studies indicate a complex interplay between TMDs, stress, depression, anxiety, and functional activities [19,20]. Symptoms of anxiety, depression, and somatization are associated with TMDs, even when not necessarily severe in frequency [21]. The prevalence of stress, depression, and anxiety is higher in TMD patients compared to healthy controls [22]. Depression and anxiety are also associated with restrictions in jaw function [23]. Yunhao Zheng showed that low income is associated with impaired jaw function via anxiety and depression in patients with TMD [24].

Although TMDs are known to cause mandibular functional limitations, several key questions regarding the relationship between specific jaw functional activities and TMDs remain unresolved. Some authors have also explored the association between TMD subgroups and the extent of mandibular functional limitation; however, inconsistencies in subgroup classifications across studies hinder direct comparisons [9,20]. Most studies have focused solely on pain-based subgroup classifications of TMDs [16]. Research investigating the association between specific TMD subgroups and individual functional activities, such as chewing tough food, chewing chicken, consuming soft food, achieving sufficient oral aperture for drinking from a cup, swallowing, yawning, speaking, and smiling, remains limited. This study aims to evaluate the degree of mandibular functional limitations across different TMD subgroups and investigate their relationship with anxiety and depression.

## Materials and methods

### Study design

This study was cross-sectional in design.

### Study setting

120 patients diagnosed with TMDs according to the DC/TMD criteria were recruited from the practice facilities of the School of Dentistry, Hanoi Medical University, Vietnam, from 2023-04-01 to 2024-07-31.

### Participants

#### Inclusion criteria.

Patients were eligible if they were over 16 years of age and underwent a standardized diagnostic process for temporomandibular disorders (TMDs) according to the *Diagnostic Criteria for Temporomandibular Disorders* (DC/TMD, Axis I) [5]. Diagnosis was based on a structured medical history interview and standardized clinical examination, including jaw range of motion (pain-free opening, maximum unassisted and assisted opening, lateral and protrusive movements), palpation of the masticatory muscles and temporomandibular joints for pain or tenderness, and assessment of joint sounds. Patients with suspected disc displacement were referred for magnetic resonance imaging (MRI), while those with suspected degenerative joint disease were referred for cone-beam computed tomography (CBCT). All examinations were conducted by dentists trained and calibrated in the DC/TMD protocol, following the recommendations of the International Network for Orofacial Pain and Related Disorders Methodology [6,25]. Only patients who had not received any form of TMD treatment prior to presentation (including occlusal splints, physiotherapy, intra-articular injections, botulinum toxin, or medication use) were eligible. Data were therefore collected at the baseline pretreatment visit.

#### Exclusion criteria.

A history of severe neurological disorders, autoimmune diseases affecting the joints and muscles, advanced malignancies, psychiatric conditions, substance abuse (including alcohol, drugs, and analgesics), facial or head trauma (especially fractures within the past 10 years), rheumatoid arthritis, psoriatic arthritis, gout, or any systemic diseases that could potentially impact the masticatory system or cause facial or jaw inflammation [23]. Patients with a history of neck and facial surgery or radiation therapy within the past 3 months or those who have received other treatments for neck or temporomandibular joint conditions were excluded. Additionally, Patients who were pregnant, taking medications known to affect the neuromuscular system, or already undergoing treatment for TMD were excluded [26–28].

### Variables and data measurement

The Jaw Functional Limitation Scale-8 (JFLS-8) is a questionnaire designed to assess the degree of mandibular dysfunction during activities such as chewing tough food, chewing chicken, eating soft food requiring no chewing, opening wide enough to drink from a cup, swallowing, yawning, talking, and smiling [1]. If a patient completely avoids an activity due to difficulty, they will circle ‘10’. If a patient avoids an activity for reasons unrelated to pain or difficulty, they will leave that item blank [5,6].

Other psychosocial factors such as GCPS 2.0, PHQ-9, GAD-7 and OBC were assessed according to the DC/TMD Axis II. The GCPS 2.0, a chronic pain assessment tool, comprises eight items measuring both pain intensity and functional limitations. The pain intensity score was calculated as the average value of responses from questions 2–4, multiplied by 10. The dysfunction score was measured by adding the points for the number of days of dysfunction and the functional interference score. The number of days of dysfunction was assessed in question 5 and converted to points: 0–6 days were converted to 0 points, 7–14 days to 1 point, 15–30 days to 2 points, and ≥31 days to 3 points. The functional interference score was calculated as the average value of responses to questions 6–8, multiplied by 10, and then converted to points: 0–29 was assigned 0 points, 30–49–1 point, 50–69–2 points, and ≥70–3 points [29–31]. The PHQ-9 was administered to evaluate depressive symptoms, consistent with DC/TMD recommendations [5,6]. Response options for each of the nine items are: not at all: 0 points; several days: 1 point; more than half the days: 2 points; nearly every day: 3 points. Total scores are categorized as follows: 0–4 (none), 5–9 (mild), 10–14 (moderate), 15–19 (moderately severe), and 20–27 (severe) [32]. Anxiety symptoms were assessed using the GAD-7 scale. Each item is scored from 0 (not at all) to 3 (nearly every day), with total scores categorized as: 0–4 (none), 5–9 (mild), 10–14 (moderate), and 15–21 (severe) [5,6]. The OBC evaluates parafunctional behaviors, summing responses to items scored from 0 to 4. Total scores of 0–16 are considered within the normal range. Scores of 17–24 are twice as frequent in individuals with TMDs, while scores ≥25 are associated with a significantly increased risk of developing TMDs [5,6]. All questionnaires were administered according to the standardized DC/TMD protocol [6]. The internal consistency reliability of the scales was evaluated using Cronbach’s alpha, with scores above 0.7 indicating acceptable reliability [33]. Pain intensity was assessed using a 100-mm Visual Analog Scale (VAS), a horizontal line anchored at 0 mm (“no pain”) and 100 mm (“worst pain”). Participants indicated their current pain intensity by marking the line, and the distance (in millimeters) from the “no pain” anchor to the mark was measured [34].

Maximum mouth opening is measured in millimeters (mm) as the distance between the incisal edges of the right mandibular and maxillary central incisors when the patient opens their mouth maximally without pain [35].

The principal investigator conducted standardized examinations and participant guidance under advisor supervision *to minimize bias during data collection.*

### Statistical analysis

Data were entered and analyzed using SPSS version 20.0 software. Post-hoc power analysis was conducted to evaluate the adequacy of the sample size. With 120 patients, the study achieved over 80% power to detect medium effect sizes (Cohen’s d = 0.50, α = 0.05). Continuous variables were summarized using means and standard deviations. The Mann-Whitney U and the Kruskal-Wallis test were used to compare means between groups for non-normally distributed data. The relationships between JFLS-8 and GAD-7, PHQ-9, VAS, OBC and GCPS were assessed using linear regression. Statistical significance was set at *p* < 0.05.

### Ethical approval

Ethical approval for this study was granted by the Hanoi Medical University Biomedical Research Ethics Committee (certificate number 858/GCN-HĐĐĐ NCYSH-ĐHYHN) on March 14, 2023. The study adhered to the STROBE guidelines, and all methods were conducted in accordance with relevant regulations. Written informed consent was obtained from all participants, with parental or legal guardian consent provided for those under 18.

## Results

### Demographic characteristics

One hundred twenty patients were included in the study, of which 85 (70.8%) were female and 35 (29.2%) were male. The mean age of the participants was 30.92 ± 12.16 years.

### JFLS-8 scores

The mean JFLS-8 score was 24.04 ± 12.54. The JFLS-8 score showed an increasing trend with age, rising from 20.42 in participants under 20 years to 27.23 in those aged 40 and older, this difference was not statistically significant. Similarly, no statistically significant difference in mean JFLS-8 scores was observed between male and female participants (p > .05).

A statistically significant difference was found in JFLS-8 scores among groups with different pain durations (p < .05). The pain-free group had the lowest JFLS-8 score (4 ± 1.73), while the chronic pain group had the highest (26.44 ± 9.64). This suggests an association between longer pain duration and greater functional limitation, as reflected in higher JFLS-8 scores (Table 1).

**Table 1:** Comparison of mean JFLS-8 scores among age, sex, duration of pain, and oral behavior groups.

Parameter	JFLS-8 (Mean ± SD)	p value
**Age**		
**<20 (n = 19)**	20.42 ± 13.03	.254*
**20-29 (n = 47)**	23.49 ± 10.85	
**30-39 (n = 32)**	24.81 ± 14.88	
**≥40 (n = 22)**	27.23 ± 11.72	
**Gender**		
**Male (n = 35)**	26.06 ± 16.74	.650**
**Female (n = 85)**	23.21 ± 10.35	
**Duration of pain**		
**No pain (n = 3)**	4.00 ± 1.73	.003*
**Acute (n = 92)**	23.77 ± 12.80	
**Chronic (n = 25)**	27.44 ± 9.80	
**OBC**		
**Normal (0–16) (n = 52)**	22.27 ± 10.91	.566*
**Risk factor (17–24) (n = 43)**	24.19 ± 11.48	
**High risk factors (25–62) (n = 25)**	27.48 ± 16.65	
**GAD-7**		
**None (n = 66)**	21.80 ± 10.92	.011*
**Mild (n = 30)**	24.50 ± 12.17	
**Moderate (n = 19)**	24.58 ± 9.95	
**Severe (n = 5)**	48.8 ± 19.28	
**PHQ-9**		
**None (n = 71)**	21.18 ± 11.20	0.001*
**Mild (n = 36)**	24.19 ± 9.37	
**Moderate (n = 7)**	30.71 ± 10.56	
**Moderately severe (n = 3)**	44.67 ± 6.66	
**Severe (n = 3)**	53.67 ± 25.54	

*Kruskal‑Wallis test, **Mann-Whitney U test.

**Abbreviations: JFLS-8** = Jaw Functional Limitation Scale 8; **OBC** = Oral Behavior Checklist; **GAD-7** = General Anxiety Disorder 7; **PHQ-9** = Patient Health Questionnaire 9.

### JFLS-8 and pain disorders groups, TMJ disorders groups

Participants with pain (myalgia, arthralgia, and combined myalgia and arthralgia) reported significantly greater difficulty in chewing tough food, eating chicken, and yawning compared to pain-free controls (p < .05). The combined pain group exhibited higher JFLS-8 scores than the pain-free group, suggesting a potential impact of combined pain on oral function, particularly mouth opening and swallowing. No significant differences were observed between groups in activities such as opening wide enough to drink from a cup, swallowing, eating soft food, speaking, and smiling, indicating that these functions remain relatively unaffected by pain. Overall, pain groups tended to exhibit higher JFLS-8 scores compared to pain-free controls (Table 2). Within the arthralgia group, chewing tough food was the most affected activity, while swallowing was the least affected. Participants with combined myalgia and arthralgia reported the greatest functional limitations across most assessed activities. Among the three pain disorder subgroups (myalgia, arthralgia, and combined myalgia and arthralgia), those with myalgia only exhibited the least functional impairment (Table 2).

**Table 2:** JFLS-8 score distribution within pain disorder groups.

	PAIN DISORDERS					
JFLS-8	No pain (Mean ±SD) n = 3	Myalgia (Mean ±SD) n = 23	Arthralgia (Mean ±SD) n = 8	Combined myalgia and arthralgia (Mean ± SD) n = 86	Total (Mean ±SD) n = 120	p value
**1, Chew tough food**	1.67 ± 0.58	6.3 ± 1.99	7.25 ± 1.91	6.88 ± 2.07	6.67 ± 2.17	**.016**
**2, Chew chicken**	0.67 ± 0.58	4.52 ± 2.23	6.13 ± 2.80	5.64 ± 2.35	5.33 ± 2.48	**.005**
**3, Eat soft food requiring no chewing**	0 ± 0	1.13 ± 1.60	0.63 ± 0.74	1.58 ± 2.22	1.39 ± 2.04	.355
**4, Open wide enough to drink from a cup**	0 ± 0	1.22 ± 1.78	1.75 ± 2.19	1.9 ± 2.45	1.71 ± 2.30	.317
**5, Swallow**	0 ± 0	0.35 ± 0.89	0.13 ± 0.35	0.85 ± 1.96	0.68 ± 1.73	.632
**6, Yawn**	1.67 ± 2.89	3.74 ± 2.18	5.13 ± 1.64	5.5 ± 2.70	5.04 ± 2.68	**.005**
**7, Talk**	0 ± 0	0.91 ± 1.86	1.5 ± 2.14	1.84 ± 2.51	1.59 ± 2.37	.155
**8, Smile**	0 ± 0	0.91 ± 1.62	1.25 ± 2.05	1.91 ± 2.62	1.63 ± 2.42	.153
**Total**	4 ± 1.73	19.09 ± 9.32	23.75 ± 9.42	26.09 ± 12.86	24.04 ± 12.54	**.003**

Kruskal‑Wallis test.

Significant differences among TMJ disorder groups were observed for mastication activities (chewing tough food and chewing chicken), opening wide enough to drink from a cup, and yawning. Conversely, these groups found no significant differences in activities such as eating soft food (not requiring mastication), swallowing, talking, and smiling. Participants with disc displacement without reduction with limited opening (DDw/oRwLO) consistently demonstrated the highest scores for masticatory and mouth opening-related activities, suggesting more pronounced mandibular dysfunction compared to other TMJ disorders groups. In contrast, participants without TMJ disorders generally presented lower JFLS-8 scores than those with TMJ disorders, particularly in chewing-related activities (Table 3).

**Table 3:** JFLS-8 score distribution within the TMDs group.

	TMJ DISORDERS							
JFLS-8	None disc displacement (Mean±SD) n = 110	DDwR (Mean±SD) n = 33	Ddw RwIL (Mean±SD) n = 45	DDw/ oRwLO (Mean±SD) n = 35	DDw /oRw/oLO (Mean±SD) n = 6	Degenerative joint disease + Subluxation (Mean±SD) n = 11	Total (Mean±SD) n = 240	p value
**1, Chew tough food**	6.52 ± 2.20	5.76 ± 2.37	6.93 ± 1.90	7.6 ± 1.63	7.67 ± 1.75	6.27 ± 2.83	6.67 ± 2.17	**.017**
**2, Chew chicken**	5.19 ± 2.44	4.55 ± 2.59	5.42 ± 2.55	6.57 ± 1.82	6.17 ± 2.64	4.36 ± 2.62	5.33 ± 2.47	**.008**
**3, Eat soft food requiring no chewing**	1.4 ± 2.19	1.18 ± 2.04	1.56 ± 2.31	1.43 ± 1.54	1.17 ± 0.98	1.27 ± 0.91	1.39 ± 2.03	.718
**4, Open wide enough to drink from a cup**	1.59 ± 2.30	0.82 ± 1.61	1.98 ± 2.55	2.54 ± 2.38	1.83 ± 2.32	1.73 ± 2.01	1.71 ± 2.29	**.024**
**5, Swallow**	0.75 ± 1.78	0.73 ± 2.04	0.89 ± 1.92	0.49 ± 1.27	0.17 ± 0.41	0 ± 0	0.68 ± 1.72	.357
**6, Yawn**	4.64 ± 2.71	4.61 ± 2.97	5.24 ± 2.53	6.23 ± 2.53	4.67 ± 1.97	6 ± 1.41	5.04 ± 2.67	**.029**
**7, Talk**	1.45 ± 2.39	1.73 ± 2.59	1.69 ± 2.63	1.57 ± 1.85	2.5 ± 2.07	1.82 ± 2.18	1.59 ± 2.36	.654
**8, Smile**	1.65 ± 2.51	1.3 ± 1.96	1.89 ± 2.71	1.77 ± 2.32	1.33 ± 2.16	1.00 ± 2.00	1.63 ± 2.41	.775
**Total**	23.17 ± 13.03	20.67 ± 11.86	25.6 ± 13.99	28.2 ± 9.40	25.5 ± 11.20	22.45 ± 9.88	24.04 ± 12.52	**.047**

*Kruskal‑Wallis test, Bonferroni-corrected Mann-Whitney U test.*

**Abbreviations: DDwR** = Disc displacement with reduction; **DdwRwIL** = Disc displacement with reduction with intermittent locking; **DDw/oRwLO** = Disc displacement without reduction with a limited opening; **DDw/oRw/oLO** = Disc displacement without reduction without limited opening; **DD** = Degenerative diseases.

### Associations with anxiety and depression

A statistically significant difference in JFLS-8 scores was observed among groups with varying levels of anxiety (p < .05). JFLS-8 scores increased with anxiety severity, ranging from 21.8 in the normal group to 48.8 in the severe group, with the severe scoring nearly twice as high as the moderate group. Additionally, a clear difference in JFLS-8 scores was also found among groups with different levels of depression (p < .05). JFLS-8 scores increased markedly with depression severity, from 21.18 in the normal group to 53.67 in the severe group. These findings indicate a strong association between anxiety, depression level, and JFLS-8 score (Table 1).

### Correlation analysis

**Fig 1:**
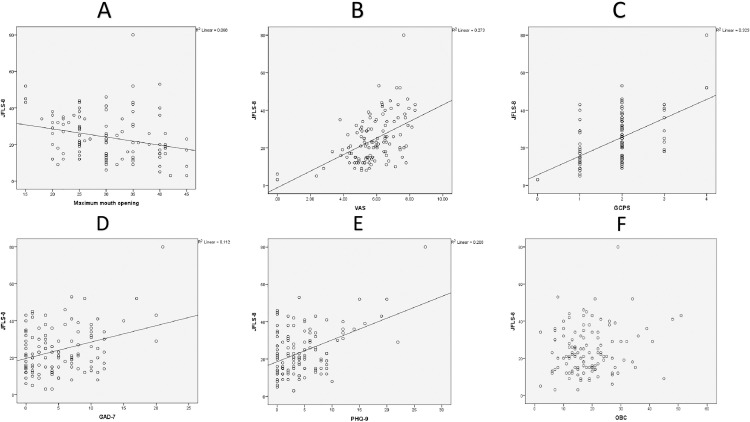
Correlation between JFLS-8 and maximum mouth opening, VAS, GCPS, GAD-7, PHQ-9 (A) JFLS-8 and maximum mouth opening (B) JFLS-8 and VAS (C) JFLS-8 and GCPS (D) JFLS-8 and GAD-7 (E) JFLS-8 and PHQ-9 (F) JFLS-8 and OBC. Abbreviations: VAS = Visual Analog Scale; GCPS = Graded Chronic Pain Scale; GAD-7 = General Anxiety Disorder 7; PHQ-9 = Patient Health Questionnaire 9; JFLS-8 = Jaw Functional Limitation Scale 8; OBC = Oral Behavior Checklist.

The JFLS-8 demonstrated significant positive correlations with VAS, GCPS, GAD-7 and PHQ-9 suggesting that impaired jaw function was associated with higher pain intensity, greater chronic pain severity, increased anxiety and elevated depressive symptoms. Fig 1B and 1C demonstrate a robust correlation between JFLS-8 and both VAS and GCPS. While the correlations between JFLS-8 and PHQ-9, GAD-7 (Fig 1D and 1E) were less pronounced, they remained positive and statistically significant. No correlation was observed between JFLS-8 and oral behaviors (Fig 1F).

## Discussion

This study emphasizes the relationship between TMD subgroups and the degree of functional impairment experienced by patients. Furthermore, it sheds light on the complex correlations between these functional impairments and psychosocial factors, notably anxiety and depression. The results corroborate existing evidence indicating that functional activities, particularly habits involving the consumption of hard and chewy foods, significantly contribute to the development and chronicity of TMD-related symptoms.

In our study, age and sex were not associated with the degree of mandibular functional limitation in TMD patients. Several authors have reported similar findings [10,36]. In contrast, Cansın Medin-Ceylan suggested that while age was not associated, female gender was found to be a risk factor for functional limitation of the jaw [19]. Yap [16] also indicated that sex is a crucial factor influencing mandibular function. Regarding sex, Kuć J found statistically significant differences in chewing tough food and smiling [18]. This finding is consistent with the established understanding that females are more susceptible to TMD problems due to their psychological and hormonal conditions.

### Jaw functional limitation and pain disorders groups

Analysis of Table 3 reveals that participants in pain groups consistently demonstrated elevated JFLS-8 scores relative to the pain-free group. This finding implies that diverse pain conditions negatively affect masticatory function and other temporomandibular joint-related functions. The extent of limitation in chewing tough food, chicken, and yawning was significantly greater in pain groups (myalgia only, arthralgia only, and combined pain) compared to the pain-free group. Yap proposed that the combined TMD group and pain-related group experience more pronounced mandibular limitations than intra-articular groups [20]. While Yap [16] et al. reported significant differences in JFLS-8 scores across various TMD subtypes (intra-articular, pain-related, and combined TMD groups, as well as groups stratified by pain presence/absence, duration, and intensity), Kim and Kim [15] found no such differences among TMD patients categorized by muscle, joint, and combined muscle-joint disorders. This discrepancy may be attributable to methodological differences, notably the use of a 20-item JFLS by Kim and Kim compared to the 8-item JFLS used in our study.

In the arthralgia-only subgroup, the most significant functional impairment was observed in the mastication of hard foods, with swallowing being the least affected function. This observation aligns with the findings of Joanna Kuc, who identified chewing tough food as the principal restriction based on the JFLS-8 assessment [18]. This phenomenon may be attributed to the biomechanical demands of mastication, wherein increased food density requires heightened bite forces and greater vertical and lateral mandibular movements, accompanied by repetitive temporomandibular joint (TMJ) translation [17]. During the consumption of hard foods, the requisite application of substantial bite force can predispose to condylar compression of the retrodiscal tissues in the open-mouth posture, consequently provoking joint pain.

Fig 1 illustrates that higher VAS scores correlate with greater mandibular functional limitation (JFLS-8). Conversely, reduced maximum mouth opening further exacerbates this limitation. Amro [37] and Ekici [10] also reached similar conclusions. There is a correlation between pain during eating and active mouth opening on the right side, meaning that if pain during eating increases, active mouth opening on the right side will decrease [37]. A strong correlation was observed between the degree of mouth opening and both pain intensity and scores on functional limitation and disability scales. Patients presenting with restricted mouth opening demonstrated significantly elevated JFLS-20 scores [10].

Yawning was significantly affected in both the arthralgia-only and combined pain groups. Consistent with our findings, Kuć et al. reported that yawning represented the second most prevalent functional restriction in their study population [18]. Yawning includes the pandiculation of the masseter, temporal, and pterygoid muscles and the prolonged contraction of the submandibular muscles. Yawning is a stereotyped phylogenetically ancient phenomenon that occurs in almost all vertebrates. As an emotional behavior and an expressive movement, yawning has many consequences [38]. Some authors highlight that yawning exacerbates pain. This is probably the effect of involuntary rapid jaw movements which affect disc position and result in quick stretch of the masticatory muscles and structures of TMJ. The consequences might be biting imbalance and loss of joint control [37]. Furthermore, morning yawning may release intra-articular adhesions with all their typical consequences [39].

Certain functions, such as swallowing, eating soft foods, speech, laughter, and adequate mouth opening for drinking from a cup, were not significantly impacted by the pain conditions, demonstrating the varying degrees to which different TMJ functions are affected (Table 3). These activities involve limited masticatory effort and muscle exertion. Impairment of these functions would indicate a severe level and intensity of pain. Pain intensity in our study was measured using the VAS. A correlation was found between VAS scores and the JFLS [9]. The maximum pain intensity recorded among participants was 8.33.

### Jaw functional limitation and TMJ disorders groups

In our study, the diagnostic process for TMJ disorder subgrouping strictly adhered to the Diagnostic Criteria for Temporomandibular Disorders (DC/TMD) guidelines. The subgroups of intra-articular disorders in our study corresponded to the subgroups in the DC/TMD. Our findings revealed that various TMJ disorders had distinct impacts on the performance of mandibular functional activities. Analysis of daily activities, including chewing tough food and chicken, achieving adequate mouth opening for drinking from a cup, and yawning, demonstrated significant variations across the TMJ disorders groups. More severe and later-stage TMJ disorders correlated with greater functional impairment. This was reflected in higher JFLS-8 scores in the group with disc displacement without reduction.

Patients with disc displacement without reduction with limited opening demonstrated significantly higher scores in masticatory and mouth opening activities, highlighting a more pronounced impact on mandibular function compared to other subgroups. This condition is characterized by an anteriorly displaced disc that cannot be recaptured by the condyle during maximal opening. Consequently, patients often report pain at maximal opening due to compression of the highly vascularized and innervated retrodiscal tissues by the condyle [39].

As a result, functional activities involving extensive interincisal opening and the application of substantial masticatory forces during the comminution of hard and tenacious foodstuffs are among the earliest to exhibit impairment. Consistent with this observation, Haketa et al. reported a lower prevalence of masticatory dysfunction in patients with myofascial pain compared to those with disc displacement, regardless of reduction status [17]. Notably, restrictions in the mastication of resilient food substances, the act of yawning, and the expression of smiling have been implicated as significant predictive factors for craniomandibular dysfunction [18]. Yuan Yao reported that individuals with anterior disc displacement with reduction exhibited significantly greater pain and functional limitations of the jaw compared to healthy controls [40]. Conversely, the investigation by Yunus Balel [9] suggests that individuals with disc displacement exhibit limitations in temporomandibular joint function, and the specific subtype of disc displacement does not differentially modulate these limitations. Although statistical analysis did not reveal significant intergroup differences in JLFS-20 scores, the subgroup with disc displacement with reduction demonstrated significantly diminished pain scores relative to both subgroups with disc displacement without reduction [9].

### Jaw functional limitation and chronic pain

We also observed a correlation between the duration of patient pain and the degree of mandibular functional limitation. The cohort experiencing chronic pain presented with the most pronounced impairment in mandibular function (Table 1 and Fig 1C). By the American Pain Society Pain Taxonomy, chronic painful TMD is defined as the persistence of nociception for a duration of ≥3 months, predicated on a reasonable time course for the biological resolution of tissue injury [41]. However, before the establishment of adaptive mechanisms within the temporomandibular joint, pain necessitates a protracted period of restricted interincisal opening and constrained mandibular excursions to prevent the exacerbation of symptoms. Concomitantly, patients frequently exhibit avoidance behaviors regarding the consumption of hard and tenacious foodstuffs to mitigate the provocation of pain. This observation is congruent with the prevailing body of evidence [19,20]. Pain can engender avoidance of temporomandibular joint mobility, masticatory function, and verbal and emotional expression, thereby precipitating functional limitations [19]. Both the intensity of pain and its interference with daily activities are recognized as key determinants of compromised jaw function, with pain-related interference exerting a more substantial effect [16]. It has been demonstrated that pain and parafunctional habits are associated with functional limitations of the temporomandibular apparatus [20].

In addition, a link between chronic pain and psychological factors such as depression and anxiety, as well as the presence of parafunctional habits, has been reported by numerous researchers. These factors, including depression, anxiety, and maladaptive parafunctional behaviors, can contribute to the worsening of chronic pain [19,42]. This interplay can establish a persistent pain cycle, posing a significant challenge for effective treatment. In contrast, Ceylan investigated the differences among groups categorized by varying durations of jaw complaints. Their findings indicated no significant difference in the extent of mandibular functional limitation across patients reporting jaw complaints for <1 year, 1–3 years, 3–5 years, 5–10 years, and >10 years [19].

### Jaw functional limitation and oral behaviors, anxiety disorders, depression

Our study findings suggest that oral behaviors did not demonstrate a significant association with mandibular functional activities in individuals with TMDs. These results are detailed in Table 1 and further explored through correlational analysis in Fig 1G. Consistent with our findings, Lovgren [43] reported a strong association between self-reported functional limitations and a screening question for frequent functional jaw disturbances rather than oral behaviors. These observations contrast with earlier research identifying a significant correlation between mandibular function and parafunctional activities [19,23]. These authors concluded that parafunctional habits were associated with functional limitations of the jaw. It has also been noted that patients with TMDs exhibit specific oral behaviors (OBs) that are linked to depression, anxiety, and jaw function [23].

Our findings further demonstrated a correlation between depression and anxiety and the extent of mandibular functional restriction in the studied patient population (Table 1, Fig 1D, 1E). Patients with TMDs who seek clinical care show a high prevalence of depression and somatization [44]. It is well-established that anxiety and depression are commonly observed in individuals experiencing chronic orofacial pain, with higher prevalence rates compared to pain-free controls in studies employing control groups, and a strong association with pain severity [45]. As pain intensifies, patients tend to avoid functional activities that provoke pain, restricting themselves to essential functions [20]. However, Yap reported that across all three TMD groups investigated, JFLS global scale and subscale scores exhibited only weak associations with somatization, depression, and anxiety scores. These findings suggest that functional jaw limitations are associated with the presence of painful TMDs but do not appear to be directly related to somatization or broader psychological distress [20].

### Limitations and future directions

While this study employed a validated self-report questionnaire based on the Diagnostic Criteria for Temporomandibular Disorders (DC/TMD), several limitations warrant consideration. First, the absence of a control group precludes the establishment of causal inferences. Second, the cross-sectional design and limited sample size restrict the generalizability of the findings to broader populations. Future research should employ larger, controlled studies. Because only untreated patients at their first consultation were included, our findings reflect pretreatment clinical status. While this reduces treatment-related confounding, the results may not fully generalize to patients who are already under active therapy. Finally, the reliance on self-reported functional limitations may have introduced response bias. To mitigate this potential bias, participants received detailed instructions regarding the questionnaires and were afforded ample time to complete them without time constraints.

## Conclusion

Significant variations in mandibular functional limitation were observed across TMD subgroups. Chewing hard food was the most significantly affected in the Arthralgia group, whereas swallowing was the least impaired. Patients with Disc displacement without reduction with a limited opening exhibited greater mandibular functional impairment compared to other diagnostic groups. The severity of these limitations demonstrated a positive correlation with levels of depression, anxiety, and chronic pain.

### Recommendation

A holistic therapeutic approach for patients diagnosed with temporomandibular disorders (TMDs) should incorporate patient education and behavioral modification strategies, with particular emphasis on minimizing the consumption of hard and tenacious foodstuffs and limiting activities that induce excessive interincisal opening, such as yawning, to reduce the probability of prolonged symptomatology and post-therapeutic recurrence. Furthermore, clinicians should address the psychosocial dimensions of the patient’s condition.

## Supporting information

S1 File Dataset. (XLSX)

S2 File Strobe checklist. (DOCX)
